# Excessive Daytime Sleepiness among the Japanese General Population

**DOI:** 10.2188/jea.15.1

**Published:** 2005-04-22

**Authors:** Yoshitaka Kaneita, Takashi Ohida, Makoto Uchiyama, Shinji Takemura, Kazuo Kawahara, Eise Yokoyama, Takeo Miyake, Satoru Harano, Kenshu Suzuki, Yuko Yagi, Akiyo Kaneko, Takako Tsutsui, Tsuneto Akashiba

**Affiliations:** 1 Department of Public Health, School of Medicine, Nihon University.; 2 Department of Psychophysiology, National Institute of Mental Health, National Center of Neurology and Psychiatry.; 3 Department of Public Health Policy, National Institute of Public Health.; 4 Department of Health Policy Science, Graduate School of Medical and Dental Science, Tokyo Medical and Dental University; 5 First Department of Internal Medicine, School of Medicine, Nihon University, Japan

**Keywords:** Sleep Disorders, excessive daytime sleepiness, general population, Japan, Snoring

## Abstract

BACKGROUND: Excessive daytime sleepiness is one of the principal symptoms of sleep disturbances, and is often associated with serious consequences including traffic and industrial accidents, decreased productivity, and interpersonal problems. However, there are few epidemiologic studies on excessive daytime sleepiness in a large scale sample targeting Japanese general population.

METHODS: The survey was performed using a self-administered questionnaire in June 2000, targeting a population randomly selected from among 300 communities throughout Japan. This questionnaire included information about sleep habits and sleep problems. Excessive daytime sleepiness measured according to a question “Do you fall asleep when you must not sleep (for example when you are driving a car)?”

RESULTS: A total of 28,714 subjects completed the questionnaire. The prevalence of excessive daytime sleepiness was 2.5% (male=2.8% and female=2.2%). Backward elimination analysis showed that the following were associated with excessive daytime sleepiness: male sex, young age, short sleep duration, subjective insufficient sleep, loss of deep sleep, disagreeable sensations in the legs, interruption of sleep by snoring or dyspnea, and feeling psychological stress. Interruption of sleep by snoring or dyspnea was the strongest associated factor (adjusted odds ratio=2.46, 95% confidence interval=1.76-3.43) of excessive daytime sleepiness.

CONCLUSIONS: These results suggest that excessive daytime sleepiness in Japanese is associated with several sleep problems. These findings may be useful in attempts to prevent excessive daytime sleepiness in the general population of Japan.

Excessive daytime sleepiness is a symptom that can result from a variety of sleep disorders,^1^ and can be distinguished from the physiological daytime sleepiness that all people may experience occasionally. As excessive daytime sleepiness can cause traffic and industrial accidents,^2^^,^^3^ its recognition is an important public health issue. However, no unified definition for excessive daytime sleepiness has yet been established because quantification is difficult, and various definitions have been used in previous studies. Because of this situation, the reported prevalence of excessive daytime sleepiness in various countries has varied considerably (0.5%-35.8%).^3^^-^^11^ For example, Hubin et al. conducted a questionnaire survey of a total of 11,354 Finnish subjects aged 33-60 years and reported a 9.0% incidence of daily daytime sleepiness.^7^ In their study, subjects who reported feeling sleepy during the daytime ‘every day’ or ‘almost every day’ were defined as having daily daytime sleepiness. Ohayon et al. carried out telephone interview surveys of a total of 4,972 British subjects aged 15 years or older and reported a 5.5% incidence of severe daytime sleepiness.^9^ They defined severe daytime sleepiness as being ‘considerably’ or ‘greatly’ sleepy during a period of at least one month.

Although a number of previous studies have examined the prevalence of excessive daytime sleepiness in a variety of subjects, there have been very few nationwide studies representing the general population, being limited to reports from Finland^7^ and Britain.^9^ Although sleep-related epidemiologic studies have been actively conducted in the United States, again there has been no nationwide study of excessive daytime sleepiness. In Japan, Liu et al. have performed an interview investigation to examine the prevalence of excessive daytime sleepiness in the general population.^12^ In Liu’s study, excessive daytime sleepiness was defined as a feeling of excessive sleepiness during the daytime “always” or “often” in two out of five choices, and it was reported that the prevalence was 14.9% in 3,030 Japanese individuals.

Although excessive daytime sleepiness is usually caused by disturbance of nocturnal sleep, it may also be unrelated to sleep at nighttime.^1^^,^^2^ It is important to elucidate the factors which relate to excessive daytime sleepiness. A number of previous studies have examined the relationship between excessive daytime sleepiness and various types of sleep problems and underlying diseases.^5^^,^^7^^-^^12^ A British study reported that symptoms of insomnia such as difficulty initiating sleep and difficulty maintaining sleep, breathing pauses or leg pain during sleep, and depressive disorder were associated with excessive daytime sleepiness.^9^ In Japan, Liu et al. reported that excessive daytime sleepiness was associated with short sleep duration, insomnia symptoms, subjective sleep insufficiency and use of sleep-enhancing medication.^12^

Liu’s study^12^ was the first epidemiologic study of excessive daytime sleepiness in a Japanese general population, and was of considerable academic value. However, it was limited by the relatively small sample size and the inclusion of few questions on sleep problems. Therefore, the actual prevalence of excessive daytime sleepiness and associated factors among the Japanese general population requires further clarification.

The present report describes an epidemiologic study of excessive daytime sleepiness in 30,000 subjects taken from the general population of Japan. The factors suspected to be associated with excessive daytime sleepiness were investigated, specifically the extent to which self-reported excessive daytime sleepiness varies according to sex, age, place of residence, feeling psychological stress and various sleep problems.

## METHODS

### Selection of Subjects

The present study was part of national survey (Active Survey of Health and Welfare), organized by the Statistics and Information Department of the Ministry of Health, Labour and Welfare. This national survey was planned to collect basic information on health and welfare. Questions concerning sleep habits and sleep problems were included in this investigation. The survey was conducted through health centers across Japan.

To ensure that the survey sample was representative of the general population, study subjects were selected from residents, 12 years of age or older, living in 300 areas. These areas were selected randomly, through stratified sampling, from approximately 824,000 areas in the National census. The survey was conducted simultaneously throughout Japan during June 2000. Part-time investigators, hired by public health centers, delivered questionnaires to the subjects and collected the completed questionnaires a few days later. Oral informed consent was obtained from subjects. Questionnaires were returned by 32,729 subjects. Because the Ministry of Health, Labour and Welfare did not publish the number of residents contacted in the target areas, the response rate could not be calculated. However, the collection rates of similar investigations carried out 3 and 4 years before were 87.1% and 89.6%, respectively.^13^^,^^14^ Because the present survey was performed using the similar methods, the response rate was estimated to be the same as that observed previously. The Minister of Health, Labour and Welfare permitted lending this survey data to us. Then, we did the analysis indicated in the following. Before analysis, 707 subjects who had submitted blank answer forms were excluded. Subjects under 20 years of age (n=3,086) were also excluded because this study was planned for adults. In addition, subjects who had not responded to the questions on sex and age were also excluded from the analysis (n=222). After these exclusions the responses from 28,714 subjects were analyzed. The sociodemographic characteristics of the analyzed subjects are shown in Table 1.

**Table 1.** Percentage of study participants and the general population classified according to sex and age.

Age(year)	Present study(2000)		Census(2000)	
	
	Male	Female	Male	Female
20-29	16%	16%	19%	17%
30-39	17%	16%	18%	16%
40-49	18%	16%	17%	16%
50-59	20%	19%	20%	19%
60-69	17%	16%	15%	15%
70+	13%	16%	12%	17%
Total	100%	100%	100%	100%

n	13599	15115	48669	52067
			(thousands)	(thousands)

Due to rounding, the percentages may not equal 100%.

### Measures

A self-administered questionnaire was developed for the present study consisting of 44 items including: (1) personal data; (2) general health status; (3) somatic and psychological complaints; (4) information on psychological stress; and (5) sleep habits and sleep problems. This questionnaire was devised by two of the authors with the appropriate official of the Ministry of Health, Labour and Welfare. The following 10 questions about sleep and stress statuses experienced during the previous month were embedded in the questionnaire:

1. Do you have difficulty falling asleep? [difficulty initiating sleep]
2. Do you wake up during the night after you have gone to sleep? [difficulty maintaining sleep]
3. Do you wake up too early in the morning? [early morning awakening]
4. Do you feel as if you have not had deep sleep when you get up in the morning? [loss of deep sleep]
5. Is your sleep interrupted by your snoring or dyspnea? [interruption of sleep by snoring or dyspnea]
6. Do you feel disagreeable sensations in your legs after you go to bed? [disagreeable sensations in legs]
7. Do you fall asleep when you must not sleep (for example when you are driving a car)? [excessive daytime sleepiness]
8. Can you take sufficiently a rest by sleep? (very sufficient/sufficient/insufficient/very insufficient).
9. How many hours do you sleep on average?
10. Do you feel dissatisfaction, worry, hardship or stress in your daily life? (very much/somewhat/hardly/never).

If the answer to the question 8 was “insufficient” or “very insufficient”, the subject was considered to have subjective insufficient sleep. In response to the question 9, a subject who reported fewer than 5 or 5-6 h sleep was considered to have short sleep duration. If the answer to the question 10, was “very much” or “somewhat”, the subject was considered to have feeling psychological stress. Difficulty initiating sleep, difficulty maintaining sleep, early morning awakening, loss of deep sleep, interruption of sleep by snoring or dyspnea, disagreeable sensations in the legs, excessive daytime sleepiness, subjective sleep insufficiency, and short sleep duration were investigated as sleep problems in this study.

### Places of residence

Places of residence of the subjects were divided into two categories: ‘urban’ and ‘rural/suburban.’ The 23 districts of metropolitan Tokyo and 12 ordinance-designated cities (Sapporo, Sendai, Chiba, Yokohama, Kawasaki, Nagoya, Kyoto, Osaka, Kobe, Hiroshima, Kitakyushu, and Fukuoka) were included in the ‘urban’ category, and any other cities and rural districts were placed in the ‘rural/suburban’ category.

### Statistical analysis

In the initial analysis, the percentages for sleep duration and subjective sleep sufficiency according to sex and age were calculated because these were generally being used as parameters of sleep, and differences were examined using the *x*^2^ test. In the second analysis, the prevalence of sleep problems including excessive daytime sleepiness was calculated by age and sex. Finally, a series of logistic regression analyses was performed to explore the association of sleep problems and psychological stress with excessive daytime sleepiness. All variables were examined initially in univariate models. Backward elimination analysis was then performed to adjust for the confounding effects of age, sex, and other factors listed in Table 5. Odds ratios were calculated from both the univariate analysis and backward elimination analysis with 95% confidence intervals. All analyses were performed using SPSS^®^ 11.0 for Windows.

## RESULTS

### Sample Characteristics

The demographic characteristics of the participants in the study are listed in Table 1. The study population and the National Census 2000 population are comparable, with similar demographics for sex and age.

### Sleep Duration and Sleep Sufficiency

Tables 2 and 3 present the percentages for sleep duration and subjective sleep sufficiency by age and sex, respectively. As can be seen in Table 2, 13.7% (males=12.9% and females=14.4%) of the study population reported sleeping for less than 6 hours. Furthermore, 33.3% (males=31.9% and females=34.5%) reported that they got very insufficient or insufficient sleep (Table 3). In both males and females, significant differences in sleep duration were observed between age classes with sleep duration increasing with age (*x*^2^ test, p<0.001). In male subjects, the highest frequency of short sleep duration was found in those aged 30-39 years, while in females, those aged 40-49 years had the highest frequency of short sleep duration.

**Table 2.** Percentage of sleep duration by sex and age.

	age(year)	n	sleep duration (hours)					

			<5	5-6	6-7	7-8	8-9	9+
Male	20-29	2147	3.6	14.3	35.7	26.2	16.3	3.8
	30-39	2163	3.4	15.3	32.6	30.7	16.0	2.1
*x*^2^=1407	40-49	2283	2.4	9.7	33.0	33.5	18.7	2.8
p<0.001	50-59	2538	1.6	9.0	29.9	34.3	21.0	4.2
d.f.=25	60-69	2061	1.9	6.9	20.9	31.6	28.7	10.0
	70+	1488	2.0	5.6	14.3	23.7	31.9	22.5
	total	12680	2.5	10.4	28.6	30.5	21.5	6.6

Female	20-29	2303	2.9	10.3	30.6	32.2	19.3	4.7
	30-39	2349	2.1	11.2	34.7	33.4	16.5	2.2
*x*^2^=1507	40-49	2409	3.2	15.3	39.9	28.6	11.8	1.2
p<0.001	50-59	2729	2.8	13.6	37.2	30.0	14.3	2.1
d.f.=25	60-69	2175	2.3	11.4	28.2	31.6	21.5	4.9
	70+	2060	2.9	7.7	19.3	23.1	26.6	20.5
	total	14025	2.7	11.7	32.1	29.9	18.0	5.5

Due to rounding, the percentages may not equal 100%.

**Table 3.** Percentage of subjective sleep sufficiency by sex and age.

	age (year)	n	subjective sleep sufficiency			

			very insufficient	insufficient	sufficient	very sufficient
Male	20-29	2149	6.5	31.6	43.9	18.0
	30-39	2168	8.4	35.4	42.4	13.8
*x*^2^=614	40-49	2288	5.9	31.3	46.3	16.5
p<0.001	50-59	2561	3.4	26.3	49.2	21.1
d.f.=15	60-69	2110	1.8	19.9	50.9	27.5
	70+	1538	1.9	15.0	51.2	31.9
	total	12814	4.8	27.2	47.2	20.9

Female	20-29	2314	4.7	30.9	47.1	17.2
	30-39	2368	6.1	35.6	45.7	12.6
*x*^2^=563	40-49	2401	7.0	35.6	45.4	12.1
p<0.001	50-59	2742	3.9	32.1	48.6	15.4
d.f.=15	60-69	2220	2.7	25.8	50.5	21.0
	70+	2137	2.3	18.0	49.1	30.6
	total	14182	4.5	30.0	47.7	17.8

Due to rounding, the percentages may not equal 100%.

Significant differences in sleep sufficiency were also observed between age classes (*x*^2^ test, p<0.001); the frequency of subjective insufficient sleep was inversely related to age. In both male and female subjects, the highest prevalence of subjective insufficient sleep by age class corresponded with that of short sleep duration.

### Prevalence of Sleep Problems

Table 4 shows the prevalence of sleep problems other than short sleep duration and subjective insufficient sleep by age and sex. In both males and females, the prevalence of difficulty maintaining sleep and early morning awakening tended to increase with age.

**Table 4.** Prevalence(%) of sleep problems by age and sex.

	Male							Female						
	
	20-29	30-39	40-49	50-59	60-69	70+	total	20-29	30-39	40-49	50-59	60-69	70+	total
Difficulty initiating sleep	17.4	14.8	13.1	12.5	13.4	14.8	14.2	21.0	18.1	13.8	21.4	23.6	21.8	20.0
Difficulty maintaining sleep	9.5	14.8	16.2	18.6	23.0	29.0	18.1	17.0	21.7	19.5	24.2	26.8	30.7	23.4
Early morning awakening	9.6	16.1	26.0	34.7	40.3	35.2	26.9	11.1	12.3	15.8	22.4	30.1	31.6	20.6
Loss of deep sleep	29.4	30.8	27.5	18.7	13.0	11.0	22.1	33.7	34.8	29.3	23.4	16.3	11.3	24.8
Interruption of sleep by snoring or dyspnea	1.5	3.3	3.3	3.1	2.5	1.8	2.6	0.8	1.0	1.5	2.2	1.7	1.5	1.5
Disagreeable sensations in legs	1.7	2.7	2.7	2.0	2.6	3.2	2.4	3.1	2.8	2.9	3.5	3.7	4.2	3.4
Excessive daytime sleepiness	4.6	3.8	3.5	2.1	1.8	0.9	2.8	4.0	2.8	3.0	1.9	0.9	0.7	2.2

n	2241	2262	2397	2710	2252	1737	13599	2400	2434	2488	2914	2424	2455	15115

The excessive daytime sleepiness was reported from 2.5% (males=2.8% and females=2.2%) of the study population. The excessive daytime sleepiness was most prevalent in young individuals (aged 20-29 years), and decreased significantly with age.

### Logistic Regression Analysis of Excessive Daytime Sleepiness - Associated Factors

The results of logistic regression analysis used to estimate the association between excessive daytime sleepiness and other variables are shown in Table 5. The odds ratios and their 95% confidence intervals are indicated. In the univariate logistic regression models, being male, young, all sleep problems and feeling psychological stress were associated with excessive daytime sleepiness. In the backward elimination analysis, being male, young, short sleep duration, subjective insufficient sleep, loss of deep sleep, interruption of sleep by snoring or dyspnea, disagreeable sensations in legs and feeling psychological stress remained significant after adjusting for all other factors. Interruption of sleep by snoring or dyspnea was the strongest associated factor of excessive daytime sleepiness. The backward elimination analysis did not reveal a significant association between excessive daytime sleepiness and difficulty initiating sleep, difficulty maintaining sleep, or early morning awakening.

**Table 5.** Logistic regression results for prediction of excessive daytime sleepiness (EDS) among the general adult population.

	Prevalence of EDS(%)	Crude	Adjusted
	
		OR (95% CI)	OR (95% CI)
Sex			
male	2.8	1.00	1.00
female	2.2	0.77 (0.67-0.90)	0.74 (0.63-0.86)

Age (year)			
20-39	3.8	1.00	1.00
40-59	2.6	0.67 (0.57-0.79)	0.72 (0.61-0.85)
60+	1.1	0.27 (0.22-0.35)	0.43 (0.34-0.55)

Places of residence			
rural/suburban	2.5	1.00	1.00
urban	2.4	0.94 (0.78-1.14)	0.85 (0.69-1.03)

Short sleep duration			
no	2.1	1.00	1.00
yes	5.8	2.82 (2.39-3.33)	1.73 (1.45-2.06)

Subjective insufficient sleep			
no	1.4	1.00	1.00
yes	5.2	3.96 (3.38-4.63)	2.37 (1.97-2.86)

Difficulty initiating sleep			
no	2.3	1.00	
yes	3.6	1.63 (1.37-1.93)	

Difficulty maintaining sleep			
no	2.4	1.00	1.00
yes	3.0	1.26 (1.06-1.49)	0.84 (0.69-1.01)

Early morning awakening			
no	2.4	1.00	
yes	2.6	1.08 (0.91-1.28)	

Loss of deep sleep			
no	1.7	1.00	1.00
yes	5.0	3.03 (2.61-3.52)	1.41 (1.19-1.67)

Interruption of sleep by snoring or dyspnea			
no	2.4	1.00	1.00
yes	7.6	3.37 (2.45-4.62)	2.46 (1.76-3.44)

Disagreeable sensations in legs			
no	2.4	1.00	1.00
yes	6.5	2.81 (2.16-3.81)	2.16 (1.59-2.93)

Feeling psychological stress			
no	1.3	1.00	1.00
yes	3.5	2.67 (2.23-3.19)	1.63 (1.34-1.97)

OR: odds ratio

CI: confidence interval

As for variables excluded by the process of backward elimination analysis, these are represented by an empty column.

## DISCUSSION

In this cross-sectional survey, representative subjects were selected randomly from the adult general population of Japan. The prevalence of excessive daytime sleepiness in this study was 2.5% (male=2.8% and female=2.2%).

In this survey, excessive daytime sleepiness was defined as sleepiness that was so severe that affected individuals fell asleep at inappropriate times, such as while driving. The use of a stricter situation than that of previous studies would explain the lower prevalence of excessive daytime sleepiness in our study. Sleepiness in the early afternoon is a natural physiological tendency, and everyone experiences occasional daytime sleepiness if their nocturnal sleep has been insufficient. Therefore, it is important to distinguish between physiological and excessive daytime sleepiness, and this point was considered seriously in our present definition of excessive daytime sleepiness. We expected excessive daytime sleepiness to be distinguished from physiological sleepiness according to this definition. The specific ‘while driving’ situation was adopted for the question, as this had often been used in other epidemiologic surveys of excessive daytime sleepiness.^10^^,^^15^

Various subjective and objective methods have been suggested for measuring excessive daytime sleepiness, but one of the most clinically useful methods is the Epworth Sleepiness Scale.^15^ This measures the probability of falling asleep in eight different situations. Unfortunately, this scale could not be used in the present study because aim of this survey was to avoid an excessive number of question items. A further study of excessive daytime sleepiness targeting the general population and using the Epworth Sleepiness Scale will be necessary in the future.

In this study, being male, young, short sleep duration, subjective insufficient sleep, loss of deep sleep, interruption of sleep by snoring or dyspnea, disagreeable sensations in legs and feeling psychological stress were all independently associated with excessive daytime sleepiness. Existences of several underlying disorders are assumed about interruption of sleep by snoring or dyspnea, disagreeable sensations in legs and feeling psychological stress. Among these, interruption of sleep by snoring or dyspnea was the greatest associated factor (adjusted odds ratio=2.46) of excessive daytime sleepiness. The association between snoring and excessive daytime sleepiness has been identified previously in several studies performed in Western countries.^16^^-^^18^ Stradling et al. found snoring to be a better predictor of excessive daytime sleepiness than direct measures of sleep apnea using overnight oximetry, and suggested that snoring alone, without nocturnal hypoxemia, may cause excessive daytime sleepiness.^16^ In the United Kingdom including Northern Ireland, Ohayon et al. found that 40% of the population reported snoring regularly, and that there was a significant association between snoring and daytime sleepiness, including drowsiness while driving.^18^ To our knowledge, the present study is the first report to indicate that snoring is a significant associated factor of excessive daytime sleepiness among the Japanese general population. The mechanisms that link snoring and excessive daytime sleepiness might include sleep apnea or other breathing difficulties that interrupt sleep. A diagnosis of obstructive sleep apnea syndrome should therefore be investigated further.

Disagreeable sensations in the legs, which are typical symptoms related to restless legs syndrome, were also found to be an associated factor of excessive daytime sleepiness (adjusted odds ratio=2.16). Restless legs syndrome was first described by Willis in 1685^19^ and was subsequently named by Ekbom in 1945.^20^ This syndrome is characterized by a desire to move the limbs, associated with paresthesias, dysesthesias, or other, sometimes indescribable, but unpleasant sensations.^1^^,^^19^^-^^22^ This desire occurs mainly in the legs, usually prior to sleep onset, and can cause difficulty in falling asleep. The pathogenesis of restless legs syndrome has not yet been elucidated. There have been few reported studies showing an association between restless legs syndrome and excessive daytime sleepiness in Japan. Therefore, this issue should be addressed in future investigations.

Many previous studies have shown that psychological factors and psychiatric diseases, particularly depression, have a significant association with excessive daytime sleepiness.^9^^,^^23^^-^^25^ In this analysis, only one item concerning a psychological or psychiatric factor was included, and this demonstrated a strong association between subjective psychological stress and excessive daytime sleepiness. This finding suggests that psychological or psychiatric disorders may be a factor underlying excessive daytime sleepiness in Japanese subjects.

In this study, several independent associated factors of excessive daytime sleepiness were identified, in particular short sleep duration and subjective insufficient sleep. The adjusted odds ratios for short sleep duration and subjective insufficient sleep in this study were 1.73 and 2.37, respectively. However, among the factors linked to insomnia symptoms, the backward elimination analysis showed that difficulty initiating sleep, difficulty maintaining sleep, and early morning awakening were not independent associated factors of excessive daytime sleepiness. There are obviously many potentially confounding factors resulting in insomnia symptoms such as sex, age, other sleep problems and feeling psychological stress, which may explain why they were not good independent predictors of excessive daytime sleepiness.

Previous studies targeting the Japanese general population reported that lifestyle, health status factors, sociodemographic factors, and somatic and psychological complaints were associated with sleep problems. Ohida et al. reported that unhealthy lifestyle-related factors such as lack of exercise and irregular eating habits, and also poor health status, were associated with short sleep duration and subjectively insufficient sleep.^26^ Doi et al. indicated that sociodemographic factors such as marital status and job status were associated with insomnia symptoms.^27^ Kim et al. showed that a number of somatic and psychological complaints, including headache, fatigue, worry, or loss of interest, were significantly associated with insomnia.^28^ In addition, it is expected that seasonal and environmental factors would be associated with sleep problems. Although it is important to examine such associations, it was not possible to do so in this study because of the limitation in the number of question items administered to the subjects. This issue should be examined in future investigations.

The present study has some limitations. First, the data on excessive daytime sleepiness were based on self-reporting, which could have biased the findings. However, several studies have indicated that self-reported data on sleep status show at least a moderate agreement with data from laboratory studies.^29^^,^^30^ Second, this study was a cross-sectional investigation and as such could not demonstrate causal directions. As the main purpose of this study is to clarify the prevalence of excessive daytime sleepiness and associated factors among the general population in Japan, and not to discuss a causal relationship between them, our goal has been achieved. Third, the questionnaire did not cover all the diseases for which an association with excessive daytime sleepiness was assumed. For instance, no question item concerning narcolepsy was set. However, it is considered that this would have had little influence on the study results because the prevalence of this disease is extremely low.^6^

In conclusion, the prevalence of excessive daytime sleepiness among the Japanese general population was 2.5%. Being male, young, short sleep duration, subjective insufficient sleep, loss of deep sleep, interruption of sleep by snoring or dyspnea, disagreeable sensations in legs, and feeling psychological stress were all independently associated with excessive daytime sleepiness, with interruption of sleep by snoring or dyspnea having the strongest association. The results suggest that excessive daytime sleepiness in Japanese is associated with several underlying disorders. These findings are important when attempting to prevent excessive daytime sleepiness in the general population of Japan.

## References

[1] El-Ad B, Korczyn AD Disorders of excessive daytime sleepiness - an update. J Neurol Sci 1998;153:192-202. 9511878 10.1016/s0022-510x(97)00291-8

[2] Roth T, Roehrs TA Etiologies and sequelae of excessive daytime sleepiness. Clin Ther 1996;18:562-76. 8879887 10.1016/s0149-2918(96)80207-4

[3] Roth T, Roehrs TA, Carskadon MA, Dement WC. Daytime sleepiness and alertness. In: Kryger MH, Roth T, eds. Principles and practice of sleep medicine, 2nd edn. Philadelphia: W.B.Saunders Company, 1994;40-9.

[4] Lavie P Sleep habits and sleep disturbances in industrial workers in Israel: main findings and some characteristics of workers complaining of excessive daytime sleepiness. Sleep 1981;4:147-58. 7256075 10.1093/sleep/4.2.147

[5] Martikainen K, Hasan J, Urponen H, Vuori I, Partinen M Daytime sleepiness: a risk factor in community life. Acta Neurol Scand 1992;86:337-41. 1455978 10.1111/j.1600-0404.1992.tb05097.x

[6] Partinen M. Epidemiology of sleep disorders. In: Kryger MH, Roth T, eds. Principles and practice of sleep medicine, 2nd edn. Philadelphia: W.B.Saunders Company,1994:437-52.

[7] Hublin C, Kaprio J, Partinen M, Heikkila K, Koskenvuo M Daytime sleepiness in an adult, Finnish population. J Intern Med 1996;239:417-23. 8642234 10.1046/j.1365-2796.1996.475826000.x

[8] Breslau N, Roth T, Rosenthal L, Andreski P Daytime sleepiness: an epidemiological study of young adults. Am J Public Health 1997;87:1649-53. 9357347 10.2105/ajph.87.10.1649 PMC1381128

[9] Ohayon MM, Caulet M, Philip P, Guilleminault C, Priest RG How sleep and mental disorders are related to complaints of daytime sleepiness. Arch Intern Med 1997;157:2645-52. 9531234

[10] Nugent AM, Gleadhill I, McCrum E, Patterson CC, Evans A, MacMahon J Sleep complaints and risk factors for excessive daytime sleepiness in adult males in Northern Ireland. J Sleep Res 2001;10:69-74. 11285057 10.1046/j.1365-2869.2001.00226.x

[11] Hara C, Lopes Rocha F, Lima-Costa MF Prevalence of excessive daytime sleepiness and associated factors in a Brazilian community: the Bambui study. Sleep Med 2004;5:31-6. 14725824 10.1016/j.sleep.2003.09.009

[12] Liu X, Uchiyama M, Kim K, Okawa M, Shibui K, Kudo Y, Sleep loss and daytime sleepiness in the general adult population of Japan. Psychiatry Res 2000;93:1-11. 10699223 10.1016/s0165-1781(99)00119-5

[13] The Ministry of Health, Labour and Welfare, Active Survey of Health and Welfare. 1997. Available at: http://www1.mhlw.go.jp/toukei/h-fukusi/gaiyo.html (Accessed July 13, 2004). (in Japanese)

[14] The Ministry of Health, Labour and Welfare, Active Survey of Health and Welfare. 1996. Available at: http://www1.mhlw.go.jp/houdou/0906/h0601-1a.html#c (Accessed July 13, 2004). (in Japanese)

[15] Johns MW A new method for measuring daytime sleepiness: the Epworth sleepiness scale. Sleep 1991;14:540-5. 1798888 10.1093/sleep/14.6.540

[16] Stradling JR, Crosby JH, Payne CD Self reported snoring and daytime sleepiness in men aged 35-65 years. Thorax 1991;46: 807-10. 1771603 10.1136/thx.46.11.807 PMC1021034

[17] Fitzpatrick MF, Martin K, Fossey E, Shapiro CM, Elton RA, Douglas NJ Snoring, asthma and sleep disturbance in Britain: a community-based survey. Eur Respir J 1993;6:531-5. 8491303

[18] Ohayon MM, Guilleminault C, Priest RG, Caulet M Snoring and breathing pauses during sleep: telephone interview survey of a United Kingdom population sample. BMJ 1997;314:860-3. 9093095 10.1136/bmj.314.7084.860 PMC2126255

[19] Willis T. The London practice of physick. London: Bassett & Cooke, 1685:404.

[20] Ekbom KA Restless legs: a clinical study. Acta Med Scand 1945;158:S1-61.

[21] Ekbom KA Restless legs syndrome. Neurology 1960;868-73. 13726241 10.1212/wnl.10.9.868

[22] Walters AS Toward a better definition of the restless legs syndrome. The International Restless Legs Syndrome Study Group. Mov Disord 1995;10:634-42. 8552117 10.1002/mds.870100517

[23] Detre T, Himmelhoch J, Swartzburg M, Anderson CM, Byck R, Kupfer DJ Hypersomnia and manic-depressive disease. Am J Psychiatry 1972;128:1303-5. 4335278 10.1176/ajp.128.10.1303

[24] Hetta J, Rimon R, Almqvist M Mood alteration and sleep. Ann Clin Res 1985;17:252-6. 3909920

[25] Benca RM. Mood disorders. In: Kryger MH, Roth T, eds. Principles and practice of sleep medicine, 2nd edn. Philadelphia: W.B.Saunders Company, 1994;899-913.

[26] Ohida T, Kamal AM, Uchiyama M, Kim K, Takemura S, Sone T, The influence of lifestyle and health status factors on sleep loss among the Japanese general population. Sleep 2001;24:333-8. 11322717 10.1093/sleep/24.3.333

[27] Doi Y, Minowa M, Okawa M, Uchiyama M Prevalence of sleep disturbance and hypnotic medication use in relation to sociodemographic factors in the general Japanese adult population. J Epidemiol 2000;10:79-86. 10778031 10.2188/jea.10.79

[28] Kim K, Uchiyama M, Liu X, Shibui K, Ohida T, Ogihara R, Somatic and psychological complaints and their correlates with insomnia in the Japanese general population. Psychosom Med 2001;63:441-6. 11382271 10.1097/00006842-200105000-00013

[29] Frankel BL, Coursey RD, Buchbinder R, Snyder F Recorded and reported sleep in chronic primary insomnia. Arch Gen Psychiatry 1976;33:615-23. 178287 10.1001/archpsyc.1976.01770050067011

[30] Hoch CC, Reynolds CF, Kupfer DJ, Berman SR, Houck PR, Stack JA Empirical note: Self-report versus recorded sleep in healthy seniors. Psychophysiology 1987;24:293-9. 3602285 10.1111/j.1469-8986.1987.tb00298.x

